# Using inertial measurement units for quantifying the most intense jumping movements occurring in professional male volleyball players

**DOI:** 10.1038/s41598-023-33056-8

**Published:** 2023-04-10

**Authors:** Ricardo Franco Lima, Ana Filipa Silva, Sérgio Matos, Henrique de Oliveira Castro, André Rebelo, Filipe Manuel Clemente, Hadi Nobari

**Affiliations:** 1 Escola Superior Desporto e Lazer, Instituto Politécnico de Viana do Castelo, Rua Escola Industrial e Comercial de Nun’Álvares, 4900-347 Viana do Castelo, Portugal; 2 Research Center in Sports Performance, Recreation, Innovation and Technology (SPRINT), 4960-320 Melgaço, Portugal; 3 Health Sciences and Human Development (CIDESD), The Research Centre in Sports Sciences, 5001-801 Vila Real, Portugal; 4 Department of Sports, Higher Institute of Educational Sciences of the Douro, 4560-708 Penafiel, Portugal; 5 Faculdade de Educação Física, Universidade Federal de Mato Grosso, Cuiabá, 78068-600 Brazil; 6 CIDEFES, Centro de Investigação em Desporto, Educação Física e Exercício e Saúde, Universidade Lusófona, 1749-024 Lisboa, Portugal; 7 COD, Center of Sports Optimization, Sporting Clube de Portugal, 1600-464 Lisboa, Portugal; 8 Delegação da Covilhã, Instituto de Telecomunicações, 1049-001 Lisboa, Portugal; 9 Department of Exercise Physiology, Faculty of Educational Sciences and Psychology, University of Mohaghegh Ardabili, Ardabil, 56199-11367 Iran; 10 Faculty of Sport Sciences, University of Extremadura, 10003 Cáceres, Spain

**Keywords:** Physiology, Engineering

## Abstract

The purpose of this study was to use an inertial measurement unit (IMU) to analyze variations in the jump outcomes concerning weekly training days, and the dependencies between the number of jumps per minute and the jump height. An experimental research design was adopted across three weeks of the final play-off of a volleyball championship. Through an IMU, the external load of seven male elite volleyball athletes of a top rating team from Portuguese 1st Division (age: 30.5 ± 3.5 years; height: 200.2 ± 6.3 cm; body mass: 93.0 ± 8.1 kg; BMI: 23.1 ± 2.3 kg/m^2^) was monitored. Repeated measures ANOVA was executed to compare the outcomes between training days. It was observed a similar density of jumps during the week. However, when comparing MD-1 to MD-2, a more significant average number of jumps per minute was observed in MD-1 (1.3 ± 0.2 vs. 1.0 ± 0.2). Additionally, a positive, large and significant correlation was registered between the number of jumps and the height of the jump. Those results highlight the benefits of the specific training, leading to greater stimulation and improvement, in a game-like context, of the stretching-shortening cycle, observed in every jump action in volleyball.

## Introduction

Volleyball is a non-invasive team sport with specific demands. The intermittent characteristics of volleyball, with high intensity and short duration, increase the relevance of monitoring each athlete with different playing roles during the training and game sessions^1,2^. Several studies have studied the importance of monitoring the training workload to achieve high performance in elite volleyball^1,3–6^. The workload in volleyball could be measured as internal and/or external load and could be defined across several parameters, such as volume, frequency, intensity, monotony, and density, among others^7,8^. In this respect, while external workload could be described as an indirect measure represented by the amount of the physical work, such as distance, velocity, number of jumps per session, which are the most common measures of external load in different studies^9–11^.

Regarding the jump load measure, it is known that there is a relationship between the match and training jump load in volleyball^12^. Furthermore, knowing that jump demands are high in elite volleyball^12^, training should be planned according to the period of the season as well as the days of the week to the match day^12^. Despite the data regarding the external workload in volleyball presented in recent studies, the technology in sports, such as the Inertial Measurement Unit (IMU) composed of an accelerometer, gyroscope, and magnetometer^13,14^ has been an important tool to help coaches and athletes to monitor the load during the training and game sessions. This kind of technology could also provide information about jump intensity and rest periods^4^. In a recent systematic review of external load in professional male volleyball^12^ results showed that, despite match and training load seeming to be similar, the jumps made according to playing position varied. Thus, the middle blocker performs the highest number of jumps^4^. The setter, according to the specific role, has a low-intensity jumps^12,15^ and the opposite, the highest ones^16^.

Moreover, the studies above analyzed the jump load and the number of jumps performed per minute. Nonetheless, no information was obtained about the density of jump load from the players across the training and game sessions. In this way, to improve the quality of monitoring the workload of elite volleyball players, it is necessary to analyze all possibilities that data could give to coaches, namely the training and match density.

Thereby, little is known about the density of jumps in volleyball, although this metric should be used to better understand the workload variance according to playing role and match result. Is in this previous thought that this study aimed to use an IMU to (i) describe the number of jumps and the jump height standardized to the time while considering the maximum and the average per minute; (ii) analyze variations of the jump outcomes in regards of weekly training days, and (iii) analyze the dependencies between the number of jumps per minute and the height of the jump.

## Methods

### Participants

Seven male elite volleyball athletes (3 outside hitters, 1 setter, 1 opposite and 2 middle blockers) of a top rating team from Portuguese 1st Division (age: 30.5 ± 3.5 years; height: 200.2 ± 6.3 cm; body mass: 93.0 ± 8.1 kg; BMI: 23.1 ± 2.3 kg/m^2^) participated in this study. The players were monitored during the championship play-off final (3 weeks of training sessions, comprehending 15 training sessions with 79,8 ± 5.7 min). The following inclusion criteria were used: (i) players did not have injuries or illness during the period of data collection; (ii) players participated, at least, in one entire set of the matches realized in the weekend and; (iii) athletes participated in the total of 15 training sessions.

All the players voluntarily participated in the study and were informed about the study’s design, implications, risks, and benefits. After receiving this information regarding the study, the players signed an informed consent. They were free to withdraw from the research if they so wished. The study was conducted in line with the international ethical guidelines for sport and exercise science research recommended by the Declaration of Helsinki^31^.

### Experimental approach

An experimental research design was adopted. To respond to our purpose, it was opted to select the 3 last training weeks of the championship due to its decision. A comparative research design tested the differences between the workload among player's roles. Players were monitored for external load in all matches and training sessions using an IMU^15^.

### Procedures

#### External load monitoring

An IMU consisting of 3-axis gyroscopes, 3-axis magnetometers, and 3-axis accelerometers (Vert® Classic, MyVert, Florida, FL, USA) was used by each player during the data collection period to determine the external load of each training session. This unit estimated the number of jumps performed and the height of each jump. The inertial sensor was placed on a belt secured to athletes’ hips after warming up and data were collected only in the beginning of technical or tactical actions (in the case of training sessions) and in the beginning of each match. This instrument is a valid tool for field-based jump load measurements and has been used in other studies^1,13,15,16^. The data were transmitted via Bluetooth to an application (MyVert Coach from IOS), making it possible to monitor all athletes in real-time. The device presents a mean error of − 2.4 cm when compared to gold-standard methods, such as force plate or video system^15^. The same IMU was always used for the same player to avoid variability between devices.

#### Training density

To obtain the training density of training sessions, each jump action was analysed according to the training session period. Thus, the number of jumps per minute and the mean height per minute across every training session were counted (from the first jump to the final jump—in training sessions). This analysis it was only possible because data extracted from the device gives information on when the jump was performed (Table 1).

**Table 1:** Information extracted from the MyVert Coach App represents one jump.

Datetime	session type	player name	height (cm)
2022–03-12 19:06:23.35	Training session	Name of the player	94

#### Statistical procedures

The descriptive statistics were analyzed using average, standard deviation and coefficient of variation (%). After confirming the data's normality, homogeneity, and sphericity, a repeated measures ANOVA was executed to compare the outcomes between training days. The post hoc test of Bonferroni was used in these cases where repeated measures ANOVA revealed significant differences (*p* < 0.05). Pearson product correlation test was executed to test the dependencies between the number and height of jumps. The magnitude of correlations was classified as follows: 0.0–0.1, trivial; 0.1–0.3, small; 0.3–0.5, moderate; 0.5–0.7, large; 0.7–0.9, very large; > 0.9 nearly perfect. The statistical treatment was conducted in the SPSS software (version 27.0.0.0, IBM, Chicago, USA) for a *p* < 0.05.

### Institutional review board statement

The study was conducted in accordance with the Declaration of Helsinki and approved by the Institutional Review Board of Escola Superior de Desporto e Lazer from Instituto Politécnico de Viana do Castelo (CE-003–2020).

### Informed consent statement

Informed consent was obtained from all subjects involved in the study.

## Results

**Figure 1:**
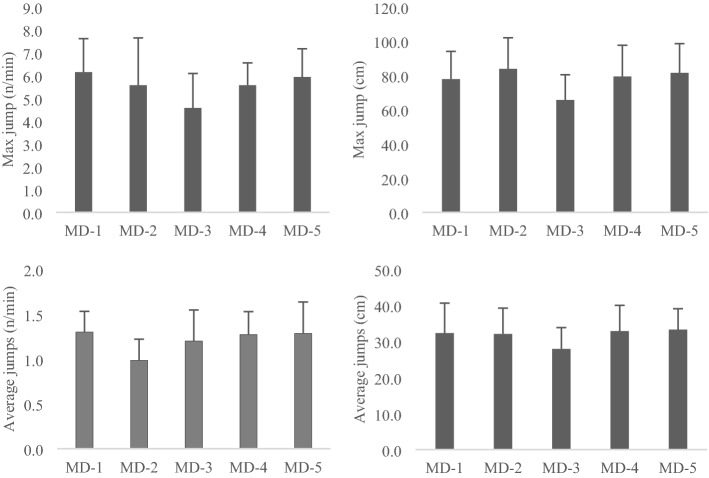
Mean and standard deviation (average of the group) of maximum values recorded for the maximal jump height, maximal jump value, average maximal jump height, maximum number of jumps per minute, and average number of jumps per minute split by training sessions.

Figure 1 displays the maximum values recorded for the maximal jump height, maximal jump value, average maximal jump height, maximum number of jumps per minute, and average number of jumps per minute.

In the MD-1 (training session the day before the match), the maximum value registered was eight jumps per minute, with a jump height of 101 cm. They performed an average of 4 jumps per minute with an average of 67 cm of jump height. The coefficient of variation over the training session period was 45% for the maximum number of jumps per minute, 34% for the maximum jump height, 59% for the average number of jumps per minute, and 47% for the average jump height.

In the MD-2 (training session two days before the match), the players achieved a maximum of 9 jumps per minute, with a maximum of 107 cm jump height. They performed an average of 4 jumps per minute with an average of 73 cm of jump height. The coefficient of variation over the training session period was 47% for the maximum number of jumps per minute, 14% for the maximum jump height, 45% for the average number of jumps per minute, and 34% for the average jump height.

In the MD-3 (training session three days before the match), the players achieved a maximum of 7 jumps per minute, with a maximum of 77 cm jump height. They performed an average of 3 jumps per minute with an average of 57 cm of jump height. The coefficient of variation over the training session period was 45% for the maximum number of jumps per minute, 14% for the maximum jump height, 49% for the average number of jumps per minute, and 37% for the average jump height.

In the MD-4 (training session four days before the match), the players achieved a maximum of 7 jumps per minute, with a maximum of 108 cm jump height. They performed an average of 4 jumps per minute with an average of 70 cm of jump height. The coefficient of variation over the training session period was 30% for the maximum number of jumps per minute, 13% for the maximum jump height, 51% for the average number of jumps per minute, and 32% for the average jump height.

In the MD-5 (training session five days before the match), the players achieved a maximum of 8 jumps per minute, with a maximum of 105 cm jump height. They performed an average of 2 jumps per minute with an average of 75 cm of jump height. The coefficient of variation over the training session period was 50% for the maximum number of jumps per minute, 39% for the maximum jump height, 47% for the average number of jumps per minute, and 46% for the average jump height.

Table 2 presents the descriptive statistics of the jump height (maximal and average) and maximal and average number of jumps per minute observed per playing position while considering the training session of the week.

**Table 2:** Descriptive statistics (mean ± standard deviation) of the athletic performance measures obtained per minute in different playing positions and weekly training sessions.

	Jumps (N max/min)	Max jump (cm)	Jumps (N average/min)	Average jump height (cm)
MD-1				
Middle blocker	7.5 ± 0.7	74.5 ± 2.1	1.3 ± 0.1	29.2 ± 5.7
Opposite	7.0 ± 0.0	94.0 ± 0.0	1.4 ± 0.0	41.5 ± 0.0
Outside hitter	5.3 ± 1.5	83.7 ± 15.0	1.1 ± 0.2	34.5 ± 9.0
Setter	5.0 ± 0.0	51.0 ± 0.0	1.6 ± 0.0	22.3 ± 0.0
MD-2				
Middle blocker	8.5 ± 0.7	80.0 ± 11.3	1.2 ± 0.1	34.3 ± 3.8
Opposite	4.0 ± 0.0	106.5 ± 0.0	1.0 ± 0.0	38.7 ± 0.0
Outside hitter	4.3 ± 0.6	101.7 ± 33.5	0.8 ± 0.1	30.2 ± 10.0
Setter	5.0 ± 0.0	57.0 ± 0.0	1.3 ± 0.0	26.4 ± 0.0
MD-3				
Middle blocker	6.0 ± 1.4	73.3 ± 4.6	1.5 ± 0.2	31.1 ± 1.0
Opposite	4.0 ± 0.0	73.0 ± 0.0	0.9 ± 0.0	29.5 ± 0.0
Outside hitter	4.0 ± 1.7	62.0 ± 21.7	1.0 ± 0.3	24.5 ± 8.5
Setter	4.0 ± 0.0	54.0 ± 0.0	1.6 ± 0.0	30.3 ± 0.0
MD-4				
Middle blocker	6.5 ± 0.7	80.5 ± 4.9	1.5 ± 0.1	34.3 ± 8.1
Opposite	6.0 ± 0.0	108.0 ± 0.0	1.3 ± 0.0	40.4 ± 0.0
Outside hitter	5.0 ± 1.0	78.3 ± 13.7	1.0 ± 0.2	32.3 ± 7.2
Setter	5.0 ± 0.0	51.5 ± 0.0	1.4 ± 0.0	24.1 ± 0.0
MD-5				
Middle blocker	7.0 ± 1.4	80.5 ± 10.6	1.6 ± 0.4	35.2 ± 3.0
Opposite	7.0 ± 0.0	92.0 ± 0.0	1.0 ± 0.0	36.6 ± 0.0
Outside hitter	5.3 ± 0.6	76.0 ± 26.2	1.2 ± 0.3	30.8 ± 8.8
Setter	6.0 ± 0.0	54.0 ± 0.0	1.6 ± 0.0	23.5 ± 0.0
Overall				
Middle blocker	8.5 ± 0.7	82.5 ± 7.8	1.4 ± 0.0	32.9 ± 3.9
Opposite	7.0 ± 0.0	108.0 ± 0.0	1.2 ± 0.0	38.6 ± 0.0
Outside hitter	6.0 ± 1.0	105.7 ± 30.0	1.0 ± 0.1	32.3 ± 7.7
Setter	6.0 ± 0.0	57.0 ± 0.0	1.5 ± 0.0	24.8 ± 0.0

Overall (excluding the training session), the middle blocker presented an 8.5 ± 0.7 n/min jump per minute, while the setter and outside hitter presented the 6.0 ± 1.0 and 6.0 ± 0.0 n/min, respectively. The opposites and the outside hitter presented 108.0 ± 0.0 and 105.7 ± 30.0 cm, respectively maximal jumps, while the setter presented a maximal jump of 57.0 ± 0.0 cm. The average of jumps per minute in the setter and the middle blocker were 1.5 ± 0.0 and 1.4 ± 0.0 n/min, respectively, while it was 1.0 ± 0.1 n/min in the outside hitter and 1.2 ± 0.0 n/min in the opposite. On the other hand, the opposite presented the average jump height of 38.6 ± 0.0 cm, and the setter a 24.8 ± 0.0 cm.

Table 3 shows the descriptive statistics of the jump outcomes per training day. Repeated measures ANOVA revealed that only significant differences between days were found in the number of average jumps per minute (F = 4.301; *p* = 0.009; $\eta_{p}^{2} \eta_{p}^{2}$= 0.418). In this case, the post hoc analysis showed that training session MD-1 conducted a more excellent average of jumps per minute than MD-2 (*p* = 0.043).

**Table 3:** Descriptive statistics (mean ± standard deviation) of the overall group's jump outcomes while organizing each day of the week.

	MD-1	MD-2	MD-3	MD-4	MD-5	F | p | Eta
Max jump (n/min)	6.1 ± 1.5	5.6 ± 2.1	4.6 ± 1.5	5.6 ± 1.0	5.9 ± 1.2	F = 2.419; *p* = 0.076; $\eta_{p}^{2} \eta_{p}^{2}$= 0.287
Max jump (cm)	77.9 ± 16.1	83.8 ± 18.1	65.6 ± 14.7	79.4 ± 18.3	81.5 ± 17.1	F = 2.557; *p* = 0.065; $\eta_{p}^{2} \eta_{p}^{2}$= 0.299
Average jump (n/min)	1.3 ± 0.2^#^	1.0 ± 0.2^#^	1.2 ± 0.3	1.3 ± 0.3	1.3 ± 0.3	F = 4.301; *p* = 0.009; $\eta_{p}^{2} \eta_{p}^{2}$= 0.418
Average jump (cm)	32.3 ± 8.3	32.0 ± 7.2	27.9 ± 5.9	32.8 ± 7.1	33.3 ± 5.8	F = 1.211; *p* = 0.332; $\eta_{p}^{2} \eta_{p}^{2}$= 0.168

Considering the training duration, it was registered a mean of 55.4 ± 10.2 min for MD-1, 66.8 ± 13.4 min for MD-2, 69.6 ± 13.5 min for MD-3, 65.4 ± 5.5 min for MD-4 and 66.8 ± 9.1 min for MD-5.

**Figure 2:**
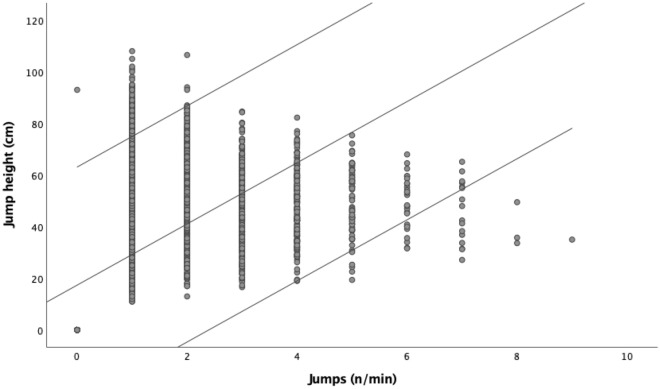
Scatter plot with jump height and jumps performed per minute.

Figure 2 presents the scatter plot with jump height and jumps performed per minute (n = 4350). The overall data showed an average number of jumps of 1.22 ± 1.4 per minute and a jump height of 31.8 ± 28.5 cm. The Pearson product correlation test revealed a positive, large and significant correlation between the number of jumps and the jump height (r = 0.573, [95% Confidence Interval: 0.553;0.593]; *p* < 0.001).

## Discussion

The present study aimed to describe the number of jumps and the jump height standardized to the time while considering the maximum and the average per minute; to analyze variations of the jump outcomes in regards of weekly training days, and analyze the dependencies between the number of jumps per minute and the height of the jump. As a whole, a similar density of jumps was observed during the week, but in opposition to the literature^17^, the MD-1 significantly stood out with a greater average number of jumps per minute when compared with MD-2 (1.3 ± 0.2 vs. 1.0 ± 0.2). However, it was registered as the lowest training duration on MD-1, showing that despite the higher values of training intensity, the training volume was adjusted. Moreover, the highest number of maximal jumps registered on MD-2 was higher than MD-1 (9 vs. 8) and were also the highest in MD-2 (107 vs 101 cm). In addition, it seems that as a specific jump, the more the player performs the attack, the better they jump, since a positive, large and significant correlation between the number of jumps and the height of jump was registered. Those results highlight the benefits of the specific training, leading to greater stimulation and improvement, in a game-like context, of the stretching-shortening cycle observed in the spike jump.

Considering the constraints imposed by the net, the ability to jump seems to play an important role in this sport. Considering that, the assessment of the types and the number of jumps that the players perform are currently monitored to understand the load applied to players. Nevertheless, the average number of jumps and their intensity and height seem essential. In this regard, in the present study was possible to observe a similar number of those highest jumps during the week, with a greater value on the MD-2, registering 9 jumps (one more than MD-1 and MD-5 and two more than MD-3 and MD-4), with 107 cm, the second highest value of the week (108 cm for MD-4). Those results follow the study of Lima et al.^16^ and Pisa et al. ^12^, which also reported a similar intensity of jumps during the week in professional volleyball players. However, Lima et al.^16^ denoted that the training sessions involved more jumps on Tuesday and Thursday (corresponding to MD-2 and MD-4), not reporting the maximum jumps. In the study of Pisa et al.^12^, no differences were observed between the frequency of jumps and the average and maximum jump height between training sessions. Compared with the present study, the results matched the maximum size reached. However, when analyzing the average number of jumps, the same was not accurate. It has been reported that a tapering effect on MD-1, lowering the training load to prepare the players for game^18,19^. However, that load reduction is generally confirmed with the number of jumps, but in the present study, although a greater average of jumps per minute were significantly higher in MD-1 compared with MD-2, the total duration of the training session, i.e. its volume, was reduced. Therefore, in the present situation, the coach also used a tapering methodology increasing density but reducing the training volume when comparing MD-1 with MD-2.

On average, volleyball professional players can perform 54–90 jumps per match and 45–128 jumps per hour of training^6,20^. However, they show different profiles regarding their game position. Under the literature^12,16^, the present study showed that the opposite, followed by outside hitter, could reach the highest jumps (108.0 ± 0.0 and 105.7 ± 30.0 cm, respectively). How those jumps are performed could affect the height reached by players^16^. For instance, the block is executed with or without a lateral approach and with a short arm swing, and the set jump is usually done without a run approach and an arm swing^6^. In opposition, in the present study, the setter presented the smallest maximal jump (57.0 ± 0.0 cm), also by the literature^13^. As observed in the present study, although the setter is used to experience the greatest demand for vertical jumps in training and games, he normally registered the lowest jumps. This is related to their demands on the game, i.e., those jumps frequently occur in the setting and that these jumps are of lesser intensity than block, attack and serve jumps^6,8,21^. Additionally, the setter has a high number of vertical jumps due to the fact that in the high level volleyball the settering, in the great majority, are performed jumping to increase the speed of the attacking actions, and the middle blockers jump in most blocking actions performed by the team as well as false attack jumps trying to confuse the opponent block.

Although the opposite and the outside hitters frequently perform jumps closer to their maximum height, the middle blockers and the setters are used to greater jumps^11,22,23^. Indeed, in a 108 training sessions analysis (27 official games and seven friendly matches of elite volleyball team),^6,13^ observed that the setter performed the highest number of jumps per session (121), followed by the middle blocker (92) and opposite hitter. Our results corroborate those studies, with a higher average of jumps per minute reporting 1.5 and 1.4 for the setter and middle blocker, respectively. Nevertheless, in a season of 32 weeks, Garcia-de-Alcaráz et al.^17^ also registered a greater number of jumps performed by the middle blocker (41.432 jumps), with an average of 83.60 ± 2.40 jumps per training session, but the opposite hitter (22.997) appeared on second place, followed by the setter (13.226). Those values will depend on the movements performed by each position, i.e., the number of jumps did not distinguish between block, attack, serve or setting jump. It was found that hitters performed an average of 32.1 ± 17.4 attack jumps and the setter 80.1 ± 44.5 setting jumps^24^, and in volleyball match, 35% of the jumps were performed in the block, 23% in the attack, 22% in the serve and 20% in the setting^25^. This results are in agreement with the study from Berriel et al.^26^ which found differences between blocking and spike jumps in professional volleyball players. However, it will always depend on the technique performed by the athletes, especially at the moment of the service and the setting, i.e., despite the men's volleyball game being characterized as more aggressive and faster, where the services and suspension settings are common, these may not have the same frequency in all teams.

An interesting result in the present study was the significant and positive correlation between the number of jumps and the height achieved in the jump. As a practical approach, coaches should be awared that, the more the player jumps, the greater stress is implemented in the musculotendinous unit. That greater stimulation of the neuromuscular coordination through training the nervous system^27^, will allow the stretch–shortening cycle (SSC), which is a lengthening movement (i.e., eccentric) quickly followed by a shortening movement (i.e., concentric)^27,28^, to react faster^29^. Moreover, according to the concept of training specificity, the effective transfer of training adaptations occurs when the training exercises match the task (e.g., testing, competition^30^); in this case, the training session matches the game situation.

The present study presents some limitations. Only one team with only seven players was analyzed for three weeks. Nevertheless, this team represents an elite and experienced men's volleyball team. Additionally, despite the results obtained, the lack of knowledge of the type of jump realized by athletes is another limitation. Therefore, the conclusions should be carefully interpreted, and more studies should be done to strengthen those results.

## Data Availability

The datasets used and/or analysed during the current study available from the corresponding author on reasonable request.
